# Fatal Progression of Mutated TP53-Associated Clonal Hematopoiesis following Anti-CD19 CAR-T Cell Therapy

**DOI:** 10.3390/curroncol30010087

**Published:** 2023-01-13

**Authors:** Lea Naomi Eder, Danilo Martinovic, Paolo Mazzeo, Christina Ganster, Justin Hasenkamp, Julia Thomson, Arne Trummer, Detlef Haase, Gerald Wulf

**Affiliations:** 1 Department of Hematology and Medical Oncology, University Medicine Goettingen, 37075 Göttingen, Germany; 2 Department of Hematology and Oncology, Städtisches Klinikum Braunschweig, 38114 Braunschweig, Germany

**Keywords:** clonal hematopoiesis, CAR-T cell therapy, clonal evolution, therapy-related neoplasia

## Abstract

We present the case of a 64-year-old man diagnosed with large B-cell lymphoma who relapsed twice after standard-of-care therapy. Due to persisting cytopenia, Next generation sequencing analysis was performed, revealing a small *TP53*-mutated clone. As a third-line therapy, the patient was treated with CAR-T cells, which resulted in complete remission. However, this treatment also led to the expansion of the *TP53*-mutated clone and therapy-related myelodysplasia with a complex aberrant karyotype. This case may serve as a paradigmatic example of clonal hematopoietic progression in a patient undergoing CAR-T cell therapy, especially in the context of a *TP53*-mutated clone.

## 1. Introduction

Early and late onset cytopenia represent common side effects of chimeric antigen receptor (CAR)-T cell therapy, to which lymphodepleting preparative therapy, prior lines of chemo- or radiotherapy, and severe cytokine release syndrome (CRS) may contribute [1,2,3,4,5,6,7,8]. Clonal hematopoiesis (CH) is frequently found in lymphoma and myeloma patients undergoing CAR-T cell therapy and has not been associated with adverse outcomes in published retrospective studies across current genetic markers for CH [9,10,11,12]. However, due to the low patient numbers in these studies, the implications of specific CH-associated genetic alterations remain unclear. Here, we present a patient with mutated *TP53* CH prior to successful anti-CD19 CAR-T cell therapy for the treatment of relapsed large B-cell lymphoma (LBCL). However, this led to the rapid progression of a mutated *TP53* hematopoietic clone, resulting in therapy-related myeloid neoplasia with complex karyotype alterations and a fatal outcome. 

## 2. Case

**Figure 1:**
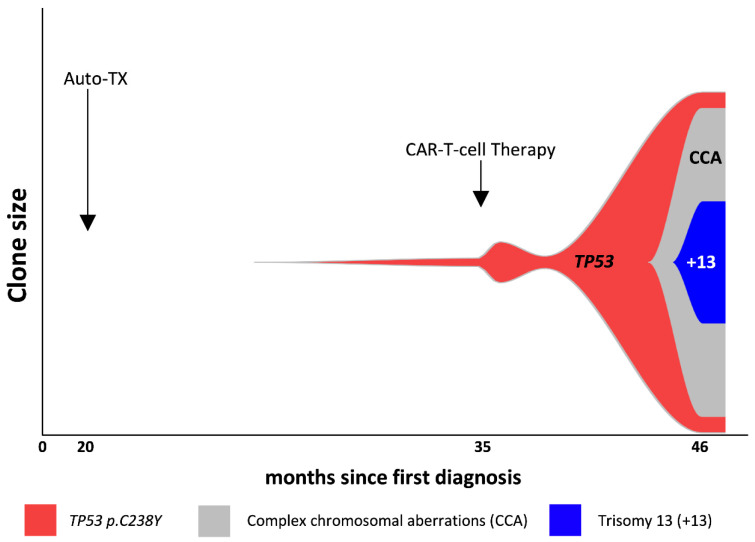
Evolution of the *TP53*-mutated clone.

A 64-year-old male was treated for LBCL stage IE with six cycles of rituximab-CHOP chemoimmunotherapy and two additional doses of rituximab mono, thereby achieving complete remission (CR). Nine months after initial diagnosis the patient relapsed and was treated with three cycles of rituximab-DHAP chemoimmunotherapy and consolidative high-dose chemotherapy (BEAM) with subsequent autologous stem cell transplantation, resulting in a second CR. However, 15 months after diagnosis the lymphoma recurred in the initially involved lymph nodes. Histological analysis confirmed relapse of the known CD19 + LBCL with synchronous cells resembling classical Hodgkin’s lymphoma (HL) cells. Prior to anti-CD19 CAR T-cell treatment against LBCL, the patient received radiation therapy targeting the involved left cervical lymph nodes, which, in turn, targeted both the nodal LBCL and HL cells as a bridging treatment. Peripheral blood (PB) analysis had shown cytopenia since the first diagnosis, which intensified after the autologous stem cell transplantation (hemoglobin 7.0 g/dL, MCV 120 fL, MCH 39.9 pg white blood cells 2.38/nL, and platelets 32/nL) without evidence of myelodysplastic syndrome (MDS) in the analyzed bone marrow aspiration or biopsy. Next-generation-sequencing (NGS) analysis from bone marrow, however, revealed a *TP53*-mutated clone (C238Y, exon 7) with a variant allele frequency (VAF) of 1.9%. No other alterations were identified using the in-house myeloid panel of 49 genes. Following the acquirement of informed consent (including with respect to reporting the course and outcome of the treatment), lymphodepletion with fludarabine and cyclophosphamide was carried out as the conditioning treatment. CAR-T cell therapy using axicabtagen-ciloleucel resulted in cytokine release syndrome (CRS) grade 2, immune effector cell-associated neurotoxicity syndrome (ICANS) grade 2, and in the metabolic CR of the lymphoma. Upon persistence of pancytopenia (hemoglobin 5.6 g/dL, white blood cells 0.55/nL, and platelets 5/nL) 1.5 months after the CAR-T cell treatment, the bone marrow analysis was repeated and showed hypoplasia without signs of dysplasia but an increase in the concentration of the *TP53* clone with a VAF of 9.8% (Figure 1). The option of an allogeneic stem cell transplantation was discussed but refused by the patient. Treatment with hypomethylating agents was excluded due to underlying cardiopulmonary disease and persisting cytopenia. Since a continuous increase in transfusion frequency—under supportive therapy including G-CSF and thrombopoietin receptor agonists—was seen during the 11 months post-CAR-T cell therapy, NGS analysis was repeated in CD34-positive blood progenitor cells isolated from PB (CD34 + pbp) using the technique developed by Martin et al. [13]. This revealed a VAF increase in the known *TP53* mutation to 83.4%, which was associated with a copy number neutral loss of heterozygosity (CN-LOH). Fluorescence in situ hybridization (FISH) showed complex aberrations with the deletion of 17p, including the *TP53* locus, in 95.5%; a *TET2* deletion in 92.9%; a 5q deletion in 98%; and a *KMT2A* deletion in 100% of the CD34 + pbp cells, reflecting a clonal evolution from a small *TP53*-mutated clone to a complex aberrant karyotype with multi-hit *TP53* mutations (Figure 1). Due to the worsening of the patient’s performance status, treatment was limited to supportive care, including IVIG substitution. We refrained from performing another bone marrow biopsy, leaving the diagnosis morphologically unconfirmed. Due to the results of the NGS and blood counts, the prognosis of a MDS would have been very poor (IPSS-R very high risk). Twelve months after CAR-T cell treatment, the patient presented to the emergency department with neutropenic fever and dyspnea and died of pneumonic sepsis despite extended antibiotic and supportive measures.

## 3. Discussion

In this study, we report a case of rapidly evolving complex karyotype aberrations after CAR-T cell treatment in a patient with bone marrow hypoplasia, originating from pre-CAR-T clonal hematopoiesis with a *TP53* mutation. The cytogenetic evolution was associated with a loss of *TP53* heterozygosity, consistent with biallelic multi-hit status, and led to a fatal outcome [14]. The *TP53*-mutated hematopoietic progenitor cells rapidly gained dominance in this patient, while blood cell regeneration from competing non-transformed stem cells was very limited due to the convergence of both CAR-T cell treatment-associated, late-onset cytopenia and pre-existing marrow hypoplasia. 

In agreement with our observations, the occurrence of MDS post-CAR-T cell treatment has been reported in several retrospective studies. In the pivotal ZUMA-1 trial utilizing axicabtagene-ciloleucel, one of 108 patients had developed MDS at 18.9 months [15], and in a single-center compilation of 31 cases from ZUMA-1 and ZUMA-9, four cases of MDS were reported after a median of 13.5 months (range: 4–26 months) following CAR-T cell treatment [16]. In another study using the same CAR backbone (FMC63-28Z) and the same conditioning treatment with fludarabine/cyclophosphamide, 2 of 43 patients developed secondary MDS at 20 and 39 months after CAR-T cell infusion, respectively [17]. Furthermore, in an anti-CD19 CAR-T cell trial employing a FMC63-4-1 BB CAR backbone, the incidence of MDS was 4% (4 in 84 cases) at a median observation time of 28.1 months [18]. The median time from CAR T-cell infusion to diagnosis of MDS was 6 months (range: 4–17 months). Interestingly, in two of the four patients that developed MDS, cytogenetic abnormalities were known prior to CAR-T cell therapy [18]. 

These cases suggest the need to screen for genetic alterations, i.e., CH, in the hematopoietic system, prior to CAR-T treatment. In three retrospective cohorts of non-Hodgkin’s lymphoma (NHL) or multiple myeloma (MM) patients treated with different CAR-T constructs, CH was detected in 34% to 48% prior to CAR-T treatment. However, CH was not associated with differences in progression-free or overall survival [9,10,11]. The most commonly affected genes were *DNMT3A*, *ASXL1*, *TET2,* and *TP53*, reflecting the findings of individuals that were not treated with CAR-T cell therapy [9,19,20]. Importantly, and similar to our case, two large retrospective studies identified 3 of 154 patients and 5 of 115 patients who developed therapy-related myeloid neoplasms during the follow-up period. In both series, two patients harbored a *TP53* sequence variant and later developed acute myeloid leukemia with *TP53* mutation [9,10]. CH has been shown to be associated with an increased risk of developing hematologic malignancies based on surveillance data from individuals without cytostatic interventions [19,20]. In situations of prolonged genotoxic stress such as repeated chemotherapy, however, the genetic and eventual clinical progression of CH might be significantly faster. This may be particularly relevant in cases with *TP53* mutations, where the loss of *TP53* heterozygosity and thereupon genetic instability occurs [14]. Interferon gamma (IFg) and Tumor Necrosis Factor alpha (TNFa) are among the important cytokines mediating CAR-T-based tumor cell killing. At the same time, however, these cytokines also exert genotoxic stress signals on hematopoietic progenitor cells [21]. We speculate that cytokine-mediated genotoxic stress may have facilitated the acquisition of further genetic alterations and karyotype evolution. This report and the case series mentioned above represent clinical observations, leaving the experimental proof of CAR-T-mediated MDS progression to appropriate in vivo [22]. 

## 4. Conclusions

The impact of genotoxic stress on the hematopoietic system associated with the cytokine and/or microenvironmental changes post-CAR-T cell therapy remain to be elucidated and the observation periods of most studies are still limited. Based on our observation together with other publications [9,10,11,12], we advocate for an early screening as well as thorough and extended follow-ups of CH with *TP53* alterations in patients receiving CAR-T cell therapy.

## Data Availability

Not applicable.

## References

[1] Maude S.L., Laetsch T.W., Buechner J., Rives S., Boyer M., Bittencourt H., Bader P., Verneris M.R., Stefanski H.E., Myers G.D. (2018). Tisagenlecleucel in Children and Young Adults with B-Cell Lymphoblastic Leukemia. N. Engl. J. Med..

[2] Fried S., Avigdor A., Bielorai B., Meir A., Besser M.J., Schachter J., Shimoni A., Nagler A., Toren A., Jacoby E. (2019). Early and late hematologic toxicity following CD19 CAR-T cells. Bone Marrow Transplant..

[3] Jain T., Knezevic A., Pennisi M., Chen Y., Ruiz J.D., Purdon T.J., Devlin S.M., Smith M., Shah G.L., Halton E. (2020). Hematopoietic recovery in patients receiving chimeric antigen receptor T-cell therapy for hematologic malignancies. Blood Adv..

[4] Chakraborty R., Hill B.T., Majeed A., Majhail N.S. (2020). Late Effects after Chimeric Antigen Receptor T Cell Therapy for Lymphoid Malignancies. Transpl. Cell. Ther..

[5] Hansen D.K., Dam M., Faramand R.G. (2021). Toxicities associated with adoptive cellular therapies. Best Pract. Res. Clin. Haematol..

[6] Schubert M.-L., Schmitt M., Wang L., Ramos C., Jordan K., Müller-Tidow C., Dreger P. (2020). Side-effect management of chimeric antigen receptor (CAR) T-cell therapy. Ann. Oncol..

[7] Sharma N., Reagan P.M., Liesveld J.L. (2022). Cytopenia after CAR-T Cell Therapy—A Brief Review of a Complex Problem. Cancers.

[8] Taneja A., Jain T. (2021). CAR-T-OPENIA: Chimeric antigen receptor T-cell therapy-associated cytopenias. Ejhaem.

[9] Miller P.G., Sperling A.S., Brea E.J., Leick M.B., Fell G.G., Jan M., Gohil S.H., Tai Y.-T., Munshi N.C., Wu C.J. (2021). Clonal hematopoiesis in patients receiving chimeric antigen receptor T-cell therapy. Blood Adv..

[10] Saini N.Y., Swoboda D.M., Greenbaum U., Ma J., Patel R.D., Devashish K., Das K., Tanner M.R., Strati P., Nair R. (2022). Clonal Hematopoiesis Is Associated with Increased Risk of Severe Neurotoxicity in Axicabtagene Ciloleucel Therapy of Large B-Cell Lymphoma. Blood Cancer Discov..

[11] Teipel R., Kroschinsky F.P., Kramer M., Kretschmann T., Egger-Heidrich K., Krüger T., Ruhnke L., Herold S., Stasik S., Sockel K. (2022). Prevalence and variation of CHIP in patients with aggressive lymphomas undergoing CD19-directed CAR T-cell treatment. Blood Adv..

[12] Uslu U., June C.H. (2022). CAR T-cell Therapy Meets Clonal Hematopoiesis. Blood Cancer Discov..

[13] Martin R., Acha P., Ganster C., Palomo L., Dierks S., Fuster-Tormo F., Mallo M., Ademà V., Gómez-Marzo P., De Haro N. (2018). Targeted deep sequencing of CD34+ cells from peripheral blood can reproduce bone marrow molecular profile in myelodysplastic syndromes. Am. J. Hematol..

[14] Bernard E., Nannya Y., Hasserjian R.P., Devlin S.M., Tuechler H., Medina-Martinez J.S., Yoshizato T., Shiozawa Y., Saiki R., Malcovati L. (2020). Implications of TP53 allelic state for genome stability, clinical presentation and outcomes in myelodysplastic syndromes. Nat. Med..

[15] Locke F.L., Ghobadi A., Jacobson C.A., Miklos D.B., Lekakis L.J., Oluwole O.O., Lin Y., Braunschweig I., Hill B.T., Timmerman J.M. (2019). Long-term safety and activity of axicabtagene ciloleucel in refractory large B-cell lymphoma (ZUMA-1): A single-arm, multicentre, phase 1–2 trial. Lancet Oncol..

[16] Strati P., Varma A., Adkins S., Nastoupil L.J., Westin J., Hagemeister F.B., Fowler N.H., Lee H.J., Fayad L.E., Samaniego F. (2020). Hematopoietic recovery and immune reconstitution after axicabtagene ciloleucel in patients with large B-cell lymphoma. Haematologica.

[17] Cappell K.M., Sherry R.M., Yang J.C., Goff S.L., Vanasse D.A., McIntyre L., Rosenberg S.A., Kochenderfer J.N. (2020). Long-Term Follow-Up of Anti-CD19 Chimeric Antigen Receptor T-Cell Therapy. J. Clin. Oncol..

[18] Cordeiro A., Bezerra E.D., Hirayama A.V., Hill J.A., Wu Q.V., Voutsinas J., Sorror M.L., Turtle C.J., Maloney D.G., Bar M. (2019). Late Events after Treatment with CD19-Targeted Chimeric Antigen Receptor Modified T Cells. Biol. Blood Marrow Transpl..

[19] Genovese G., Kähler A.K., Handsaker R.E., Lindberg J., Rose S.A., Bakhoum S.F., Chambert K., Mick E., Neale B.M., Fromer M. (2014). Clonal Hematopoiesis and Blood-Cancer Risk Inferred from Blood DNA Sequence. N. Engl. J. Med..

[20] Jaiswal S., Fontanillas P., Flannick J., Manning A., Grauman P.V., Mar B.G., Lindsley R.C., Mermel C.H., Burtt N., Chavez A. (2014). Age-Related Clonal Hematopoiesis Associated with Adverse Outcomes. N. Engl. J. Med..

[21] Yan B., Wang H., Rabbani Z.N., Zhao Y., Li W., Yuan Y., Li F., Dewhirst M.W., Li C.-Y. (2006). Tumor Necrosis Factor-α Is a Potent Endogenous Mutagen that Promotes Cellular Transformation. Cancer Res..

[22] Côme C., Balhuizen A., Bonnet D., Porse B.T. (2020). Myelodysplastic syndrome patient-derived xenografts: From no options to many. Haematologica.

